# Adhesion of Respiratory-Infection-Associated Microorganisms on Degradable Thermoplastic Composites

**DOI:** 10.1155/2009/765813

**Published:** 2009-04-06

**Authors:** Teemu Tirri, Eva Söderling, Minna Malin, Matti Peltola, Jukka V. Seppälä, Timo O. Närhi

**Affiliations:** ^1^Department of Prosthetic Dentistry and Biomaterials Science, Institute of Dentistry, University of Turku, 20520 Turku, Finland; ^2^Laboratory of Polymer Technology, Department of Chemical Technology, Helsinki University of Technology, 02150 Espoo, Finland; ^3^Department of Otorhinolaryngology - Head and Neck Surgery, Turku University Central Hospital, Turku, 20520 Turku, Finland; ^4^Clinic of Oral Diseases, Turku University Central Hospital, Turku, 20520 Turku, Finland

## Abstract

The purpose of this study was to evaluate bacterial adhesion and early colonization on a composite consisting of bioactive glass (BAG) particles and copolymer of *ε*-caprolactone/D,L-lactide. Materials were incubated with suspensions of both type strains and clinical isolates of *Streptococcus pneumoniae, Haemophilus influenzae*, and *Pseudomonas aeruginosa* for 30 minutes (adhesion) and 4 hours (colonization). Clear differences exist in the microorganisms' ability to adhere on the experimental materials. However, the presence of BAG particles does not inhibit bacterial adhesion, but early colonization of the materials with *P. aeruginosa* was inhibited by the addition of 90–315 *μ*m BAG particles.

## 1. Introduction

Tissue integrating biomaterials can be utilized in various applications in head and neck and craniomaxillofacial surgeries such as the sinus augmentations and obliterations, repair of fractured orbital floor, and occluding nasal septum perforations. Bioactive glass (BAG) plates and granules are currently available for clinical use for these applications [1–5]. Good biocompatibility and osteopromotive properties of bioactive glasses are well known, which make them interesting materials for reconstructive surgery. However, glass itself is a brittle material and it cannot be easily shaped during the surgery. Composites of BAG and biodegradable polymers may offer better handling properties without losing glasses bioactive function [6, 7].

A composite of copolymer of poly(*ε*-caprolactone and D,L-lactide) and BAG (S53P4) was recently developed. The composite degrades via hydrolysis and it forms hydroxyapatite layer on its surface in simulated body conditions [8, 9]. The composite is thermoplastic and it can be shaped during the surgery. Materials' osteoconductive capacity has been proven in experimental animal study [7]. Thus, the composite of poly(*ε*-caprolactone and D,L-lactide) and BAG is a potential new material for surgical corrections of tissue defects in craniofacial area.

Biofilm forms usually on implant surfaces within few hours when they are exposed to an environment where they are continuously bathed in bacteria-containing fluids. Bacterial colonization can either be beneficial or destructive for the implanted device [10]. Materials that enhance biofilm formation may cause chronic infections in the implantation site. No data is available about early colonization of polymer composites containing BAG. In principle, in the upper respiratory tract area, there is a risk to bacterial colonization of implants. The present study aimed to evaluate the adhesion and colonization reflecting early biofilm formation of clinically important head and neck area pathogens *Streptococcus pneumoniae, Haemophilus influenzae*, and *Pseudomonas aeruginosa* [11, 12] on a composite of copolymer of poly(*ε*-caprolactone and D,L-lactide) and BAG.

## 2. Materials and Methods

### 2.1. Composite Substrates

Bioactive thermoplastic composites were made by incorporating 60 wt% of bioactive glass granules (BAG) (S53P4, Vivoxid Ltd., Turku, Finland) into the copolymer of poly(*ε*-caprolactone and D,L-lactide) through blending [8]. The composition of the BAG by wt% was SiO_2_ 53%, Na_2_O 23%, CaO 20%, and P_2_O_5_ 4%, while the particle size was 90–315 *μ*m (C90) and <45 *μ*m (C45). Copolymer without BAG was used as a control (C). Round test specimens (surface area 78.5 mm^2^, thickness 2 mm) for bacterial cultivation were punched out of the cast composite plates. Specimens were packed and sterilized with *γ*-radiation (dose 25 Gy, Gammaster, Waageningen, The Netherlands). A representative sample of the specimens was characterized prior to the microbial cultivation by measuring their surface roughness and wetting properties. Wetting performance was determined by contact angle measurement.

### 2.2. Microorganisms

The following microorganisms were used in the experiments: *S. pneumoniae* ATCC 49619, *S. pneumoniae* clinical isolate 1186; *H. influenzae* ATCC 49247, *H. influenzae* clinical isolate 1258; *P. aeruginosa* ATCC 27853, *P. aeruginosa* clinical isolate 1218. The clinical isolates were kind gifts from Dr. Erkki Eerola, Department of Microbiology, University of Turku, Finland.

### 2.3. Cultivation of the Microorganisms

For the adhesion experiments the microorganisms were cultured in Brain Heart Infusion medium (BHI; Difco Laboratories, Mich, USA). *H. influenzae* and *P. aeruginosa* were grown overnight at 37°C, producing log-phase cells with the inoculum used. For *H. influenzae* the medium was supplemented with *β*-nicotinamide adenine dinucleotide (10 mg/L) and hemin (20 mg/L) according to Kuo et al. [13]. The fast-growing *S. pneumoniae* was grown in BHI supplemented with heat-inactivated fetal calf serum (10% v/v; Promocell, Heidelberg, Germany). It was first grown overnight whereafter an inoculum was transferred to fresh medium in the morning and the cells were cultured for 3–4 hours resulting in log-phase cells. The cells were harvested by centrifugation, washed once with saline (0.9% NaCl w/v) and suspended in saline to an optical density of appr. 0.25 at the wavelength 660 nm. The *S. pneumoniae* cell suspension was prepared as described before but the optical density of the suspension was adjusted to appr. 0.5. The cell densities were chosen based on preliminary adhesion experiments showing in scanning electron microscopy (SEM) even distribution of the cells on the material surfaces with abundant space for surface colonization.

### 2.4. Adhesion Tests

The cell suspensions were prepared for the adhesion experiments as described above. The adhesion experiments were essentially performed as described earlier [14]. Each tested disc was incubated with 1.5 mL of the cell suspension at room temperature for 30 minutes using gentle rolling (The Coulter Mixer, Luton, UK) in a 14 mL capped plastic test tube with an inner diameter of 16 mm (Falcon, BD Biosciences, Bedford, Mass, USA). The mixer was tilted in a 15-degree angle to ensure that the materials were covered by the cell suspension at all times. After the rolling the material was rinsed gently 3 times in 50 mL saline. The cells adhering to the two flat surfaces of each disc were carefully scraped from the surfaces in 900 *μ*L of transport medium (Tryptic Soy Broth, Difco Laboratories), each side of the disc in a separate vial. Three applicators dipped into fresh transport medium before the procedure (Quick-Stick, Dentsolv AB, Sweden) were used to scrape the cells from one side on the disc, the brush ends of the applicators were cut into the transport media. In preliminary experiments increasing the number of scrapings did not increase the cell yield. Vortexing removed efficiently the cells from the ends of the applicators. The experiments were performed with 3–4 replicates and repeated at least once.

For the enumeration of cells on the disc surfaces as colony-forming units (CFU), the microbe samples collected from the surfaces were thoroughly Vortexed and grown on agar plates after serial dilutions of the samples. *H. influenzae* was grown on chocolate agar, *S. pneumoniae* and *P. aeruginosa* on blood agar overnight at 37°C in air supplemented with 7% CO_2_.

In some experiments, the materials were subjected to fixation followed by SEM as described in what follows.

### 2.5. Early Surface Colonization

For testing early surface colonization the materials were, after the 30 minutes rolling in the bacterial suspensions described earlier, dipped in 50 mL saline to remove cells not attached to the surfaces. The materials were then transferred to fresh growth media described in Section 2.3. The culturing was performed using rolling in 1.5 mL of growth medium in the tube used in the adhesion experiments at 37°C for 4 hours. The numbers of cells on the material surfaces were determined as described in Section 2.4. The pHs of the growth media were determined at the end of the incubation to ensure that the pH of the medium was not affected by the material, the cells growing on the material surface or shed to the medium.

The early colonization experiments were performed with 3–4 replicates. The experiments were repeated at least once.

### 2.6. SEM

For the SEM examinations the samples were fixed for 5 minutes (2% glutaraldehyde and 2% formaldehyde in phosphate-buffered saline, pH 7.4), rinsed once in distilled water and dried in an ascending ethanol series: 50% EtOH for 5 minutes, 70% EtOH for 10 minutes, two times 96% EtOH for 10 minutes, and absolute EtOH for 5 minutes. Finally the specimens were sputter coated with gold and examined with SEM (Model JSM 5500, JEOL Ltd., Tokyo, Japan).

### 2.7. Statistical Analysis

Statistical analysis was done with StatView and Graphics Program (SAS institute, Cary, NC, USA). Differences in the mean numbers of the microbes (CFUs) harvested form the experimental materials were tested with an ANOVA after logarithmic transformation. Post Hoc comparisons were made with Fisher's PLSD test after the *F* test for equal means was found to be significant at *P* < .05 level.

## 3. Results

Ra value of the samples with small glass particles was lower than that of samples with big glass particles (0.46 ± 0.08 *μ*m versus 0.51 ± 0.11 *μ*m). Contact angle of the samples with small glass particles was also smaller than that with big glass particels (46° ± 12° versus 68° ± 5°).

Clear differences were observed in bacterial adhesion and early stages of surface colonization of all experimental materials when using the type strains. *H. influenzae* (ATCC 49247, clinical isolate 1258) adhered significantly better on the copolymer C when compared to *P. aeruginosa* (ATCC 27853, clinical isolate 1218; mean difference 0.944, *P* < .0001) and *S. pneumoniae* (ATCC 49619, clinical isolate 1186; mean difference 0.430, *P* < .05). Significant difference was also noticed between *S. pneumoniae* and *P. aeruginosa* (mean difference 0.514, *P* < .01).

**Figure 1:**
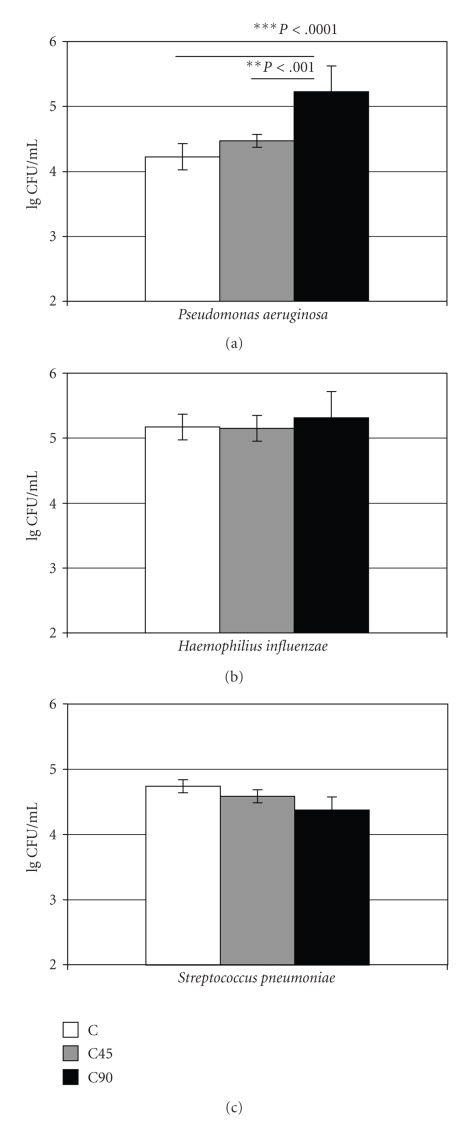
Attachment of (lg CFU/mL) *P. aeruginosa* (ATCC 27853)*, H. influenzae* (ATCC 49247), and *S. pneumoniae* (ATCC 49619) on the experimental materials C, C45, and C90.

Adhesion of *P. aeruginosa* was significantly higher to C90 as compared to the C (mean difference 1.000; *P* < .001). The adhesion was also higher to C90 as compared to C45 (mean difference 0.756, *P* < .01). The adhesion of *H. influenzae* and *S. pneumoniae* did not differ between the tested materials (Figure 1).

Similar results were obtained with the type strains and the clinical isolates of the microorganisms in the adhesion experiments. Thus the surface colonization experiments were performed with the type strains only.

**Figure 2:**
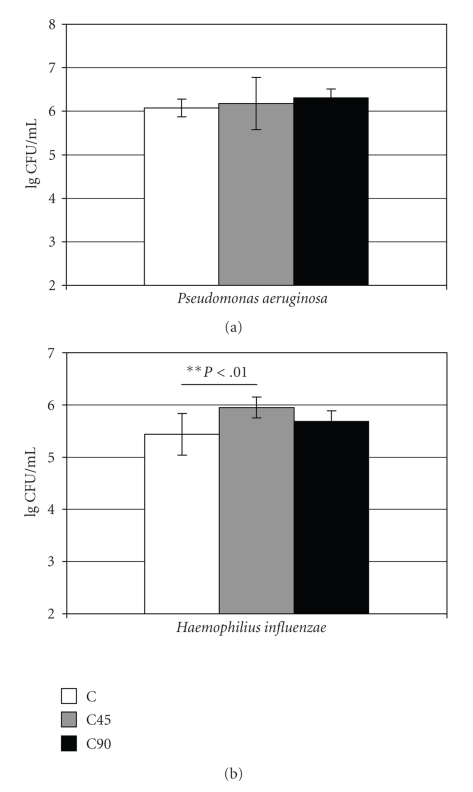
Early colonization (lg CFU/mL) by *P. aeruginosa* (ATCC 27853) and *H. influenzae* (ATCC 49247) on the experimental materials C, C45, and C90.

*P. aeruginosa* showed rapid surface colonization during the culturing and was superior to *H. influenzae* in this respect (Figures 1 and 2). *S. pneumoniae* was not able to colonize the material surfaces in the experimental conditions used and the number of viable cells harvested from the materials after the 4-hour culturing actually decreased (*P* < .001).

For *P. aeruginosa* the differences observed in the cell colonization evened up after the 4-hour culturing indicating that C90 inhibited surface colonization (Figure 2): no differences among the materials were observed although significantly more viable cells had adhered on C90 during the 30 minutes adhesion. *H. influenzae* showed surface colonization of all materials (Figure 2). No colonization inhibition was observed, actually, slightly higher numbers of cells were harvested from the C45 than the C discs after the 4-hour culturing.

Presence of BAG in the material had no effect on the final pH of the culture media. No differences among the experimental materials were noticed in this respect. No visible turbidity indicating growth in the medium was observed after the 4-hour culturing.

### 3.1. SEM

**Figure 3:**
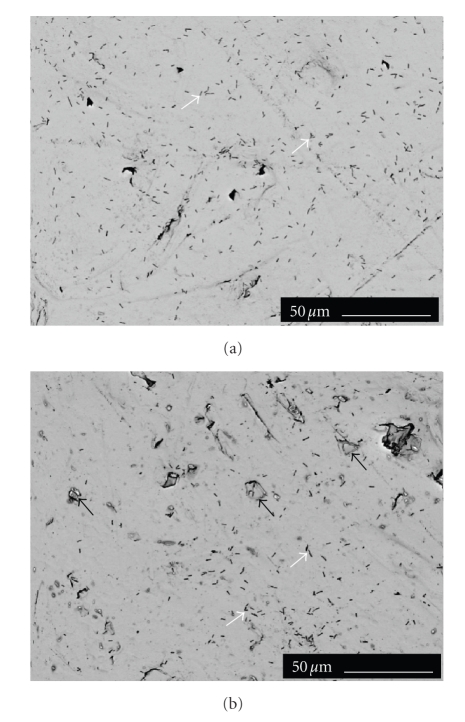
SEM image of the *P. aeruginosa* (ATCC 27853) attachment on the composite (a) and on the C90 disc (b) after 30-minute immersion (white arrows). Bioactive glass granules are visible on the surface of C90 material (black arrows). Images are inverted into negatives to show the attached microbes.

The SEM examinations verified the results obtained by culturing the cells harvested from the material surfaces. Roughness of the sample surfaces varied according to the size of BAG particles incorporated into the copolymer matrix. The smoothest surface was noticed on the copolymer surface C, while the material C90 had the roughest surface texture. Microbes seemed to attach evenly on the materials' surfaces, but no microbes were detected directly on the surfaces of the exposed BAG granules as is shown in Figure 3 for *P. aeruginosa* after 4 hours of culturing. The rough surface of C45 and C90 appeared to favor adhesion of *P. aeruginosa* but this was not seen for *H. influenzae* and *S. pneumoniae*. The results of the SEM analyses appeared to be in agreement with the quantitative CFU assessments of the cells from the material surfaces.

## 4. Discussion

Primary adhesion between bacteria and implant devices is mediated by nonspecific interactions [10]. Before the adhesion, implant surface is conditioned by the adsorption of water and proteins, which alters its original surface properties. Thus, in clinical environment bacterial adhesion and subsequent biofilm development is strongly determined by materials ability to adsorb fluids from their surroundings.

In the present study all the experimental materials consisted of poly(*ε*-caprolactone/D,L-lactide) or its composites with BAG. Due to the polycaprolactone blocks the copolymer itself is hydrophobic and has relatively poor wettability [8]. The presence of BAG increases water adsorption, but the adsorption occurs mainly through the interface of the BAG and copolymer matrix. As formed, all BAG granules are immediately embedded in the copolymer matrix and even near the surface granules are covered by a thin polymer film. The samples used in this study were prepared by casting, which resulted in different topographies on the specimen surfaces. Casting was chosen in order to achieve circumstances which resemble clinical situation after material application. Due to the thermoplastic nature of the materials, a thin polymer skin covers the embedded galss granules even after the material is shaped into its desired form. Grinding and polishig would have resulted in more uniform surface texture, but thermoplastic nature and softness of the material prevented using this method.

In this study the hydrophobic nature of copolymer did not prevent the adhesion of microorganisms as all tested microbes attached on the experimental materials. Copolymer samples without BAG showed relatively smooth surface texture. However, the presence of BAG particles induced irregularities on the sample surfaces, which increased the surface area of all the samples containing BAG.

The purpose of this work was to study attachment of *H. influenzae, S. pneumoniae, and P. aeruginosa* to the composites as well as net accumulation of the bacteria to the materials during the 4-hour exposure to the growth medium. The growth media did not show turbidity after the experiments, thus cells shed from the material surfaces should not have interfered with the experiments. In this study, we used scraping of the cells and not labelling of them as earlier [14] since we wanted to collect both sides of the discoid material specimens for separate assessment. This enabled also the assessment of CFUs and SEM detection from the same specimen. The repeatability of the scraping method was good as judged by the small standard deviations of the replicates. Thus the differences in bacterial adhesion and early colonization are related with the differences in the composition and surface topography of the tested materials.

In the previous studies BAG has shown to possess direct antimicrobial properties against *S. mutans*, *Porphyromonas gingivalis*, *Actinobacillus actinomycetemcomitans*, and *Actinomyces naeslundii* [4, 15, 16] and even against *Candida albicans* [17]. In these studies a clear antimicrobial effect has been noticed when BAG has been used in the particulate form. In this form, BAG's active surface area is large and it increases significantly the pH of the aqueous environment on its surrounding. It is likely that the main antimicrobial effects of BAG are related to its reactivity and capability to increase the pH.

In our study all the materials were in solid discoid form. Although BAG particles are reactive and capable of changing pH in the vicinity of the composite surface [18] it did not prevent bacterial attachment in this study. The attachment of *P. aeruginosa* was increased by the presence of large BAG granules, most probably due to larger surface area, while the attachment of *H. influenzae* and *S. pneumoniae* was influenced by the chemical nature of the material. In a previous study *H. influenzae* and *S. pneumoniae* attached poorly on BAG plates [4, 19]. Embedding BAG in the copolymer apparently hinders the surface reactions of the BAG particles since only the granules close to the composite surface are exposed to dissolution in the short-term immersion. Despite that, in our study the presence of BAG appeared to inhibit colonization, reflecting early biofilm formation, as the expected values based on bacterial attachment did not increase. As expected, *P. aeruginosa* could colonize on the materials, and also *H. influenzae* was able to grow on the materials and stay attached to them, while *S. pneumoniae* did not colonize at all. The differences in the inhibiton of colonization among the studied microorganisms is propably related with the surface reactivity of the experimental materials. Prevention of *P. aeruginosa* colonization was especially clear on substrates containing large BAG particles.

Within the limitations of this study it can be concluded that composites of poly(*ε*-caprolactone and D,L-lactide) and BAG do not inhibit the adhesion of *S. pneumoniae, H. influenzae* or *P. aeruginosa* to the copolymer. However, the presence of BAG seems to inhibit their colonization. This is especially clear for *P. aeruginosa*, which is known to colonize easily on surfaces. One of the major drawbacks in the use of biomaterials is the occurrence of biomaterials centred infections [20] and, thus, any method to prevent them is beneficial. The findings in the present study suggest that in terms of bacterial colonization the composite of poly(*ε*-caprolactone and D,L-lactide) and BAG is a promising material for medical devices in head and neck and craniomaxillofacial applications.
